# Risk factors for falls with severe fracture in elderly people living in a middle-income country: a case control study

**DOI:** 10.1186/1471-2318-8-21

**Published:** 2008-08-26

**Authors:** Evandro SF Coutinho, Astrid Fletcher, Katia V Bloch, Laura C Rodrigues

**Affiliations:** 1 Department of Epidemiology, National School of Public Health, Oswaldo Cruz Foundation, Rio de Janeiro, Brazil; 2 Non-Communicable Disease Epidemiology Unit, Department of Epidemiology and Population Health, London School of Hygiene & Tropical Medicine, London, UK; 3 Institute of Studies in Public Health, Federal University of Rio de Janeiro, Rio de Janeiro, Brazil; 4 Infectious Diseases Epidemiology Unit, Department of Infectious and Tropical Diseases, London School of Hygiene & Tropical Medicine, London, UK

## Abstract

**Background:**

Fracture after falling has been identified as an important problem in public health. Most studies of risk factors for fractures due to falls have been carried out in developed countries, although the size of the elderly population is increasing fast in middle income countries. The objective of this paper is to identify risk factors for fall related to severe fractures in those aged 60 or more in a middle-income country.

**Methods:**

A case-control study was carried out in Rio de Janeiro-Brazil based general hospitals between 2002–2003. Two hundred-fifty hospitalised cases of fracture were matched with 250 community controls by sex, age group and living area. Data were collected for socio-demographic variables, health status and drugs used before the fall. A conditional logistic regression model was fitted to identify variables associated with the risk of fall related severe fracture.

**Results:**

Low body mass index, cognitive impairment, stroke and lack of urine control were associated with increased risk of severe fall related fractures. Benzodiazepines and muscle relaxants were also related to an increased risk of severe fractures while moderate use of alcohol was associated with reduced risk.

**Conclusion:**

Although the association between benzodiazepines and fractures due to fall has been consistently demonstrated for old people, this has not been the case for muscle relaxant drugs. The decision to prescribe muscle relaxants for elderly people should take into account the risk of severe fracture associated with these drugs.

## Background

About one third of the population aged 65 or more suffers at least one fall a year, of which 5 to 10% result in severe injuries [1-4]. More than 90% of hip fractures are the result of a fall [5,6]. Falling and the frequency of falls increases exponentially with age [1,3-5,7]. Injuries resulting from falls incur costs for health providers, social services, patients and their families [6-8].

An active research agenda exists with a focus on prevention [2]. Cognitive impairment, low body mass index and certain medications such as benzodiazepines have been consistently associated with severe injuries from falls [5,9-12]. Data on the proportion of fall-related injuries attributable to each of these factors is sparse and it is likely that their relative contribution varies from one setting to another.

Most studies of risk factors for fractures due to falls have been carried out in developed countries, although the size of the elderly population is increasing fast in middle income countries. In Brazil the proportion of people over 60 doubled from 4.1% in 1940 to 8.6% in the year 2000, and it is expected to reach 14% in 2025 [13-15]. Little is known about the frequency, circumstances, risk factors and consequences of falls in Brazil. Perracini & Ramos [16] examined risk factors for any kind of fall in a cohort of elderly community residents and Rozenfeld et al [17] did a cross-sectional investigation in a recreational facility for the elderly. Coutinho & Silva [18] carried out a case-control study with hospital controls investigating the association between drugs used in the previous 24 hours and the risk of severe fracture after falling.

We conducted a study to investigate a range of health related factors associated with falls leading to hospitalisation due to fractures among elderly people.

## Methods

### Design

A case-control study was carried out in the city of Rio de Janeiro, Brazil.

### Participants

Two hundred and fifty cases were selected from patients aged 60 or more, admitted to five state hospitals (two are university hospitals and three are funded by the local government) with a severe fracture following a fall, between 2002–2003. A fall was broadly defined as an episode in which a person came to rest on the ground or floor and severe fracture was the one leading to hospital admittance. These hospitals admitted about 50% of all cases of severe fracture in the people aged 60 or over in Rio de Janeiro and were in different geographic areas of the city. The State Health System in the city covers about 70% of the adult population.

Two hundred and fifty controls were individually matched for sex, age (± 2 years) and neighborhood (residence of cases and controls in the same block). No elected case refused to participate in the study. Selection of controls was carried out using a systematic procedure starting from the case address, with the direction around the block pre-defined by chance. We did not find more than one eligible control per household. Twenty-one people who filled the criteria for being a control did not want to take part in the study and were replaced.

### Data collection

The interviewers visited the hospitals everyday to look for new cases. All individuals aged 60 or more admitted to the hospital to treat a fracture were approached and those reporting a fall as the cause of the injury were asked to participate in the study. Interviews took place at hospital (cases) and home (controls) through standard questionnaires applied by trained interviewers of both sexes. All interviewers had a university degree. The questionnaires consisted of a common set of questions and information was obtained from cases, controls and relatives. These included socio-demographic characteristics, circumstances of the fall, self-reported health status before the fall, information on drug use 24 hours and 15 days before the fall (for cases) or before the home visit (for controls), height, weight, current diseases (self reported) and cognitive impairment (evaluated by an adapted translation of the "Short Care" [19] which was validated in Brazil by Veras et al [20]); and history of falls and fractures in the previous 12 months. The present study used the information of the drugs taken in the previous 24 hours, categorised in 21 groups [see Additional file 1].

### Sample size

Without considering the matching, a sample size of 500 individuals would allow to identify an odds ratio of 2 for an exposure of 13% among controls (confidence level = 0.95 and power = 0.80).

### Statistical analysis

First, an unadjusted analysis (except for the matching variables) was carried out for all socio-demographic and health related variables using conditional logistic regression. In this level all variables with p-value less than 0.25 were selected for multivariate analysis [21]. Second, a multivariate conditional logistic regression model including those variables was fitted to the data. At this stage, variables with p-value equal or less than 0.05 were maintained in the model. Third, variables with p-value larger than 0.25 in the first stage (univariate analysis) were entered in the model and retained if their p-value were equal or less than 0.05. This last stage was carried out aiming to reduce the chance of excluding important predictors for severe fall related fractures.

Variations in the magnitude of the odds ratios after removing those variables, and multiplicative interactions between drugs and clinical variables were also investigated. The statistical significance of the interaction terms was investigated comparing the models with and without the interaction term through the likelihood ratio test.

We used literature-based categories for BMI and cognitive impairment for ease of interpretation.

Complementary analysis comparing means used Kruskal-Wallis test as data were either asymmetrical or variances were not homogeneous.

All interviewed people signed an informed consent term. The study was approved by the ethical committee of the National School of Public Health – Oswaldo Cruz Foundation.

## Results

All 250 cases and 250 controls recruited took part in the study. The interval between the fall and the interview did not exceed 48 hours, although some additional information could have been obtained latter. The great majority were women and about half the individuals were aged between 70–79 years old (Table 1). Due to matching, cases and controls had a similar distribution for age, with mean age for cases 75.5 years old (sd: 8.2) and 75.3 years old (sd: 7.7) for controls. Widowhood was the most frequent marital status for both groups, but the proportion of divorced was higher among cases than controls. The large majority of those interviewed were not living alone. More than 40% did not complete elementary education. Less than 15% were working before the fall.

**Table 1:** Distribution of socio-demographic variables among cases (n = 250) and controls (n = 250).

Variable	Cases n (%)	Controls n (%)
Sex		
Female	55 (78.0)	55 (78.0)
Age group (years)		
60–69	59 (23.6)	56 (22.4)
70–79	118 (47.2)	127 (50.8)
80–89	61 (24.4)	57 (22.8)
90 and more	12 (4.8)	10 (4.0)
Marital status		
married	73 (29.2)	82 (32.8)
widowed	116 (46.4)	127 (50.8)
divorced	23 (9.2)	8 (3.2)
never married	38 (15.2)	33 (13.2)
Living alone		
no	201 (80.4)	196 (78.4)
yes	39 (15.6)	47 (18.8)
Institution	10 (4.0)	7 (2.8)
Educational level		
none + elementary incomplete elementary	104 (41.6)	110 (44.0)
.level one (about 5 years)	83 (33.2)	84 (33.6)
.level two (about 4 years)	37 (14.8)	30 (12.0)
secondary (about 3 years)	20 (8.0)	15 (6.0)
university	6 (2.4)	11 (4.4)
Working before the fall		
Yes	34 (13.6)	28 (11.2)

Seventy percent of the falls resulting in severe fracture occurred between 6:00 am and 6:00 pm, with a similar proportion in the morning and in the afternoon. Most falls took place at home (67%), and this proportion increased with age. The most commonly fractured bone was the femur (72%) followed by arm/forearm (19%). Two cases had vertebral fracture (2.7%) and eleven (4.4%) had more than one bone fractured. Ninety nine per cent of the cases had to undergo surgical procedures.

Health related factors with a level of significance less than 0.25 in their univariate association with fracture (matched analysis) are presented in table 2. Increased odds ratios were observed for low BMI, low blood pressure, dizziness, diabetes, cognitive impairment, history of stroke, lack of urine control, poor vision, limit in carrying activities of daily living (ADL), fall in the previous 12 months, use of antidepressants, benzodiazepines, muscle relaxants and cerebral vasodilators while reduced odds ratios were observed for poor health status, regular use of alcohol, calcium supplement and calcium channel blockers. Most benzodiazepines were long acting, and the most frequently prescribed was bromazepan. Almost all prescribed muscle relaxants were carisoprodol.

**Table 2:** Distribution of health related variables^1 ^among cases and controls, odds ratios^2 ^(OR), 95% confidence intervals (CI) and p-values (cases = 250, controls = 250).

Variables	Cases n (%)	Controls n (%)	OR (95% CI)	P value^1^
BMI – kg/m^2^				
25 or more	85 (34.1)	118 (47.2)	reference	< 0.01
20–24.9	107 (43.0)	104 (41.6)	1.23 (0.72–2.11)	
less than 20	57 (22.9)	28 (11.2)	3.31 (1.49–7.37)	
Health status				
excellent	27 (10.8)	43 (17.2)	reference	0.01
good	121 (48.4)	126 (50.4)	1.79 (0.85–3.76)	
fair	90 (36.0)	61 (24.4)	1.82 (0.76–4.37)	
poor	12 (4.8)	20 (8.0)	0.31 (0.08–1.27)	
Dizziness	70 (28.0)	57 (22.8)	1.33 (0.60–2.21)	0.17
Low blood pressure	15 (6.0)	3 (1.2)	5.00 (1.45–17.27)	0.01
				
Diabetes	52(20.8)	39 (15.6)	1.42 (0.90–2.25)	0.14
Cognitive impairment^3^	64 (28.7)	27 (10.8)	3.64(2.02–6.58)	<0.01
Stroke	28 (11.2)	8 (3.2)	4.33 (1.78–10.53)	<0.01
				
Lack of urine control	69 (27.6)	30 (12.0)	3.05 (1.82–5.12)	<0.01
Poor vision^4^	21 (8.8)	9 (3.6)	2.63 (1.16–5.88)	0.02
Limited in carrying ADL^5^	146 (58.4)	113 (45.2)	1.85 (1.24–2.70)	<0.01
Current use of alcohol				
not used	198 (79.2)	161 (64.4)	reference	< 0.01
less than once a week	35 (14.0)	44 (17.6)	0.79 (0.40–1.55)	
at least once a week	17 (6.8)	45 (18.0)	0.42 (0.17–1.02)	
Fall in the previous 12 months	94 (37.8)	79 (31.6)	1.34 (0.91–1.98)	0.14
Antidepressant^6^	8 (3.2)	3 (1.2)	2.67 (0.71–10.05)	0.15
				
Benzodiazepine^6^	44 (17.6)	19 (7.6)	2.56 (1.44–4.57)	<0.01
Ca channel blocker^5^	22 (8.8)	41 (16.4)	0.47 (0.27–0.84)	0.01
Ca supplement	5 (2.0%)	12 (4.8)	0.42 (0.15–1.18)	0.10
Diuretics^6^	35 (14.0)	48 (19.2)	0.68 (0.42–1.10)	0.12
Muscle relaxant^6^	21 (8.4)	8 (3.2)	5.33 (1.55–18.30)	<0.01
				
Cerebral Vasodilators^6^	27 (10.8)	14 (5.6)	2.08 (1.05–4.15)	0.04

1. Selection criteria for this table was univariate p-value < 0.25. P-values for the 2 × n comparisons

2. matched by sex, age group and neighbourhood. OR estimated using conditional logistic regression

3. adapted translation of the "Short Care"

4. unable to identify someone at the opposite side of the room

5. unable to perform at least one of the following activities on his(her) own: use of public transport, drive, walk short distances, eat own meals, dress up, take medication, comb, go up and downstairs, take shower, cut nails, control urine.

6. in the previous 24 hours

Osteoporosis, Parkinson disease, epilepsy, high blood pressure, use of angiotensin converting enzyme (ACE) inhibitors, antihistamines, analgesics, antiacids, alpha and beta-adrenergic blockers, nitrates, non steroidal anti-inflamatory drugs, digoxin, calcium and vitamin D supplements did not reached the pre-defined level of 0.25 significance [see Additional file 2].

The average total number of drugs were 2.2 (sd = 1.47) in cases and 2.1 (sd = 1.48) among the controls. The difference was not statistically significant (p = 0.43).

When variables presented in table 2 were entered in a multivariate conditional logistic model, diabetes, high blood pressure, rheumatism, poor vision and use of diuretics had significance levels over 0.05 and were dropped from the multivariate model. Table 3 presents the final model. Body mass index equal or less than 20 kg/m2, cognitive impairment, previous stroke and lack of urine control were associated with increased incidence of severe fall related fractures while use of alcohol at least once a week was associated with reduced incidence. Concerning the use of drugs in the previous 24 hours, benzodiazepines and muscle relaxants were related to an increased risk of severe fractures while calcium channel blockers were associated with a reduced risk. The highest odds ratio (approximately 5 fold) was observed for use of muscle relaxants and history of stroke, although confidence intervals were large. No effect modification was observed for the variables included in the final model.

**Table 3:** Association of health related variables and severe fall related fractures. Adjusted^1 ^odds ratios (OR), 95% confidence intervals (CI) and p-values (cases = 250, controls = 250)

Variables	OR (95% CI)	P value^2^
BMI		
25 or more	reference	
20–24.9	1.18 (0.71–1.96)	0.63
less than 20	3.43 (1.64–7.17)	<0.01
Cognitive impairment	2.19 (1.09–4.41)	0.03
Stroke	5.27 (1.31–21.20)	0.02
Lack of urine control	3.16 (1.42–7.03)	<0.01
Current use of alcohol		
not used	reference	
less than once a week	0.71 (0.38–1.33)	0.29
at least once a week	0.40 (0.18–0.89)	0.02
Benzodiazepine	2.22 (1.07–4.58)	0.03
Ca channel blocker	0.40 (0.19–0.86)	0.02
Muscle relaxant	4.42 (1.02–19.21)	0.04

1. Each variable is controlled for the other ones in the table plus self-reported health status and activities in daily living using conditional logistic regression.

2. P-values for each variable category compared to the reference level.

Model fit statistics: McFadden's R^2 ^= 0.297, McFadden's Adj R^2 ^= 0.206, Count R^2 ^= 0.777, Likelihood-ratio χ^2 ^_(14 df) _= 91.485, p < 0.001

## Discussion

Most of the cases of severe fracture due to falling in the present study were female. Most fell at home between 6:00 am and 6:00 pm and the great majority of the fractures affected the femur and the arm/forearm. Risk factors identified were low body index mass, cognitive impairment, stroke, lack of urine control, use of benzodiazepine and muscle relaxants.

Our study in a middle-income country setting agrees with some previously reported associations with fracture due to fall observed in developed countries: low body index mass [9,22,23], cognitive impairment [5,22], stroke [9,10], lack of urine control [24-26].

In the present study regular users of alcohol ("at least once a week") were at a reduced risk of severe fractures. Findings for alcohol have not been consistent. Peel et al [27] found a reduced risk of fall-related fracture for moderate alcohol intake while other authors have found the reverse: higher risk of falls leading to fracture associated with higher use of alcohol [10,28]. "J" shape patterns in alcohol use have been observed for outcomes such as cardiovascular disease, with non drinkers or those with very low intakes having higher risks compared to those with mild or moderate drinking patterns. In our study, those who reported using alcohol at least once a week rarely made use of alcohol more than twice a week. It is possible that people drinking at least once a week were healthier than those not drinking. Although we attempted to control for this by the inclusion of other health related variables in the model, we cannot exclude the possibility of residual confounding.

As in our investigation, previous studies have identified an effect of benzodiazepines on the risk of fall-related fractures [12]. Hartikainen et al [29] carried out a systematic review of 29 studies that reported the association between the use of medicines and the risk of falls or fall-related fractures among people aged 60 or more. Nine of them were case-control studies matched by sex and age like our investigation. The authors concluded that central nervous system drugs, mainly psychotropic drugs, were associated with an increased risk of these accidents,

Many drugs commonly used by elderly people have not been systematically studied as risk factor for falls [29]. An important and novel result from our study was the association between the use of muscle relaxants in the last 24 hours and severe fracture due to falling. The odds ratio was very high (OR = 4.42) although the 95% confidence interval was wide (1.02–19.21). To the best of our knowledge, there is only one study that reported this empirical association among the elderly. French et al [30] used database information to investigate the relationship between registered primary diagnosis of fracture and previous use of some drugs. The authors found that those registered with fracture were prescribed muscle relaxants 1.4 times more than controls (those with non-specific chest pain). This value is much lower than the one we found, but it is difficult to compare these findings as the study designs were quite different.

That muscle relaxants can cause falls is biologically plausible: these drugs are recognized to cause weakness, drowsiness, sedation and anticholinergic effects [31]. Data on the use of muscle relaxants by elderly people, especially for extended periods, are limited but such studies that have been done reported usage by: 3% of the 60 and over population in Rio de Janeiro-Brazil [32], 0.77% of the 60 and over population in the USA [33], and 1.2% of the 75 and over age group in Finland [34]. Although muscle relaxants are recommended for short-term treatment of back pain, Dillon et al [33] reported a mean length of use of 2.1 years in the USA; 44.5% of users referred use for more than a year. Although it is generally acknowledged that the use of muscle relaxant may be inappropriate and hazardous in the elderly [31,35], the figures quoted above show that their use and their long term use remains a problem. It is likely that usage figures will be higher in places where there is easy access to medications over the counter, commonly in low and medium income countries such as Brazil. The 2002 criteria for potentially inappropriate medication use in older adults [31] does not mention explicitly the risk of falling and suffering a fracture in its evaluation of miorelaxants; the only group of drugs for which concern with falls is mentioned is long acting benzodiazepines

In contrast to previous studies we did not find a significant association between visual impairment [9,16,36,37] and diabetes [28,38] with fall related fracture. We did not measure visual acuity but relied on self report and there may have been under-reporting leading to dilution of effect. In the case of diabetes, finding an association with falling may be influenced by the proportion of those with neurological and foot problems. Ottenbacher et al [38] found that the association between diabetes and hip fracture particularly for those taking insulin.

Our study showed an unexpected inverse association between the use of calcium channel blockers (CCB) and the occurrence of severe fall related fracture. Two systematic reviews [29,39] did not find any association between these variables. We cannot exclude the possibility that this finding was due to residual confounding of self-reported health status. CCB and angiotensin converting enzyme inhibitors (ACEI) were the most reported antihypertensives. Although the proportion of controls taking CCB and ACE was the same (16%), the average total number of drugs referred by the first group was 3.0 while by the second group it was 3.5 (p = 0.02). This suggests that users of CCB could be healthier than those to which ACEI were prescribed.

The study had some limitations. Most variables were self reported and, in some of the interviews, information was provided or added by relatives that were in the hospital (for cases) or at home (for controls). This could lead to an unknown degree of misclassification of exposures. Moreover, cognitive impairment was evaluated after the fall, and we cannot be sure about the influence of the accident on mental state.

On the other hand, our study has some strengths. There were no refuses among cases and only few among controls and ascertainment of cases was likely to be high as severe fracture will be hospitalised. Controls were selected from same population as cases. Moreover, the study was done in a low income population from a middle-income country, a setting rarely reported for studies on fall related fractures

## Conclusion

What causes falls and fractures in the elderly is an important question as the size of the elderly population is increasing fast in middle income countries. Our study identifies some similar factors and a few differences, including an important role for miorelaxant drugs which are prescribed over the counter in many countries. We studied fractures leading to hospitalisation and it is possible that muscle relaxants are also associated with less severe falls. Urgent and immediate attention is necessary to confirm and quantify the risks as to inform increased control of these drugs in elderly people.

## Competing interests

The authors declare that they have no competing interests.

## Authors' contributions

ESFC had full access to all of the data in the study and takes responsibility for the integrity of the data and the accuracy of the data analysis. Study concept and design: ESFC, LCR and AF. Acquisition of data: ESFC. Analysis and interpretation of data: ESFC, LCR and AF. Drafting the manuscript: ESFC, LCR, AF and KVB. Statistical analysis: ESFC and LCR. Study supervision: ESFC. All authors read and approved the manuscript.

## Pre-publication history

The pre-publication history for this paper can be accessed here:



## Supplementary Material

Additional file 1 Categories and ATC codes for the drugs used in the last 24 hours. The table provided presents the Anatomical Therapeutic Chemical Code for the drugs investigated. Click here for file

Additional file 2 Variables with p-value greater than 0.25 (univariate analysis). The table provided presents the odds ratios and 95% confidence intervals for the variables that did not reach statistical criteria in the preliminary analysis for inclusion in the multivariate analysis. Click here for file
