# Supplementary material for: Risk factors for falls with severe fracture in elderly people living in a middle-income country: a case control study
Source: BMC Geriatr. 2008 Aug 26;8:21. doi: 10.1186/1471-2318-8-21 (PMC2532993; doi:10.1186/1471-2318-8-21)
Supplement: Additional file 1 — Categories and ATC codes for the drugs used in the last 24 hours. The table provided presents the Anatomical Therapeutic Chemical Code for the drugs investigated. [file 1471-2318-8-21-S1.doc]

Additional file 1. Categories and ATC* codes for the drugs used in the last 24 hours.

| Drug – category | ATC codes |
| --- | --- |
| Angiotensin-converting enzyme (ACE) inhibitors | C09 |
| Antiacid | A02 |
| Antihistaminic | R06 |
| Alpha-adrenergic blocker | C02 |
| Antidepressant | N06A |
| Analgesic | N02B |
| Beta-adrenergic blocker | C07 |
| Benzodiazepine | N05B |
| Calcium supplement | A12A |
| Ca channel blocker | C08 |
| Collirium (for glaucoma) | S01E |
| Decongestionant (systemic) | R01B |
| Diuretics | C03 |
| Digitalis | C01A |
| Laxant | A06 |
| Muscle relaxant | M03 |
| Nitrates | C01D |
| Non steroidal anti-inflammatory drugs (NSAIDs) | M01A |
| Vitamin D | A11C |
| Cerebral Vasodilators | N07C |
| Other | - |

*[Anatomical Therapeutic Chemical Code](http://pt.wikipedia.org/wiki/ATC) – World Health Organization
