# Supplementary material for: Risk factors for falls with severe fracture in elderly people living in a middle-income country: a case control study
Source: BMC Geriatr. 2008 Aug 26;8:21. doi: 10.1186/1471-2318-8-21 (PMC2532993; doi:10.1186/1471-2318-8-21)
Supplement: Additional file 2 — Variables with p-value greater than 0.25 (univariate analysis). The table provided presents the odds ratios and 95% confidence intervals for the variables that did not reach statistical criteria in the preliminary analysis for inclusion in the multivariate analysis. [file 1471-2318-8-21-S2.doc]

Additional file 2. Variables with p-value greater than 0.25 (univariate analysis).

| Variable | OR^*^ (95% CI) |
| --- | --- |
| Married (*vs* non married) | 0.80 (0.51-1.23) |
| Educational level  illiterate  4 years  8 years  11 years  University degree | 1  0.87 (0.49-1.56)  1.08 (0.54-2.18)  1.17 (0.50-2.75)  0.43 (0.12-1.51) |
| Living alone | 0.80 (0.51-1.27) |
| Working before the fall | 1.28 (0.73-2.27) |
| High blood pressure | 0.81 (0.56-1.17) |
| Parkinson disease | 1.75 (0.51-5.98) |
| Rheumatism | 0.86 (0.60-1.23) |
| Osteoporosis | 1.30 (0.78-2.14) |
| Angiotensin-converting enzyme (ACE) inhibitors | 0.89 (0.55-1.43) |
| Antiacid | 1.33 (0.56-3.16) |
| Antihistaminic | 2.00 (0.18-22.06) |
| Alpha-adrenergic blocker | 0.60 (0.22-1.65) |
| Collirium (for glaucoma) | 1.33 (0.30-5.96) |
| Decongestionant (systemic) | 1.00 (0.06-15.00) |
| Digoxin | 0.80 (0.31-2.03) |
| Laxant | 0.33 (0.03-3.20) |
| Nitrates | 1.00 (0.37-2.66) |
| Non steroidal anti-inflammatory drugs (NSAIDs) | 1.08 (0.51-2.29) |
| Vitamin D | 0.50 (0.04-5.51) |

* matched by sex, age group and neighbourhood. OR estimated using conditional logistic regression.
